# South Asian maternal and paternal lineages in southern Thailand and the role of sex-biased admixture

**DOI:** 10.1371/journal.pone.0291547

**Published:** 2023-09-14

**Authors:** Wipada Woravatin, Mark Stoneking, Metawee Srikummool, Jatupol Kampuansai, Leonardo Arias, Wibhu Kutanan

**Affiliations:** 1 Department of Biology, Faculty of Science, Khon Kaen University, Khon Kaen, Thailand; 2 Department of Evolutionary Genetics, Max Planck Institute for Evolutionary Anthropology, Leipzig, Germany; 3 Biométrie et Biologie Évolutive, UMR 5558, CNRS & Université de Lyon, Lyon, France; 4 Department of Biochemistry, Faculty of Medical Science, Naresuan University, Phitsanulok, Thailand; 5 Department of Biology, Faculty of Science, Chiang Mai University, Chiang Mai, Thailand; 6 Research Center in Bioresources for Agriculture, Industry and Medicine, Chiang Mai University, Chiang Mai, Thailand; 7 Centre for Linguistics, Faculty of Humanities, Leiden University, Leiden, The Netherlands; 8 Department of Biology, Faculty of Science, Naresuan University, Phitsanulok, Thailand; Xiamen University, CHINA

## Abstract

Previous genome-wide studies have reported South Asian (SA) ancestry in several Mainland Southeast Asian (MSEA) populations; however, additional details concerning population history, in particular the role of sex-specific aspects of the SA admixture in MSEA populations can be addressed with uniparental markers. Here, we generated ∼2.3 mB sequences of the male-specific portions of the Y chromosome (MSY) of a Tai-Kadai (TK)-speaking Southern Thai group (SouthernThai_TK), and complete mitochondrial (mtDNA) genomes of the SouthernThai_TK and an Austronesian (AN)-speaking Southern Thai (SouthernThai_AN) group. We identified new mtDNA haplogroups, e.g. Q3, E1a1a1, B4a1a and M7c1c3 that have not previously reported in Thai populations, but are frequent in Island Southeast Asia and Oceania, suggesting interactions between MSEA and these regions. SA prevalent mtDNA haplogroups were observed at frequencies of ~35–45% in the Southern Thai groups; both of them showed more genetic relatedness to Austroasiatic (AA) speaking Mon than to any other group. For MSY, SouthernThai_TK had ~35% SA prevalent haplogroups and exhibited closer genetic affinity to Central Thais. We also analyzed published data from other MSEA populations and observed SA ancestry in some additional MSEA populations that also reflects sex-biased admixture; in general, most AA- and AN-speaking groups in MSEA were closer to SA than to TK groups based on mtDNA, but the opposite pattern was observed for the MSY. Overall, our results of new genetic lineages and sex-biased admixture from SA to MSEA groups attest to the additional value that uniparental markers can add to studies of genome-wide variation.

## Introduction

Located on the Malay Peninsula, Southern Thailand shares borders with Central Thailand to the North, the Andaman Sea to the West, the Gulf of Thailand to the East and Malaysia to the South. Most southern Thai people speak languages belonging to the Tai-Kadai (TK) and Austronesian (AN) language families. The KhonTai (TK) and ThaiMalay (AN) account for ~66% and 33% of the population, respectively, whereas the Maniq, one of the minor groups in the region, speak an Austroasiatic (AA) language. Maniq is one of the indigenous peoples of Southeast Asia (SEA) and probably descend from mid-Holocene hunter-gatherers [1–3], since they carry ancient genetic lineages [4, 5]. Overall, there is evidence of a long history of human occupation of Southern Thailand [6], as attested by the diversification of population subsistence patterns, cultural artifacts of Paleolithic hunter-gatherers associated with the Hòabìnhian archaeological tradition dated to ~27–38 thousand years ago (kya) [7], and human remains at Moh Khiew Cave in the Krabi Province (dated to ~26–8 kya) [8–10].

As in other areas of SEA, the shift from hunter-gatherer to farming lifestyles in the Malay Peninsula probably happened during the Neolithic Period ~5–4 kya [11, 12]. An admixed genetic portrait between the deeply diverged East Eurasian lineage and East Asians was proposed for Neolithic people who had genetic connections with some contemporary SEA AA-speaking populations [2, 13]. Therefore, AA languages probably were predominant in Mainland Southeast Asia (MSEA) during the Neolithic Period, and subsequently fragmented due to the influence of later expansions during the Bronze/Iron Age ~2.5–2 kya that are associated with present-day TK and AN speakers. In addition to migrations from East Asia (EA) to MSEA, vestiges of interactions between South Asia (SA) and MSEA are also found during the late Bronze Age to Iron Age [11]. Maritime trade networks stretched from China through SEA to India and the Mediterranean region, which allowed SEA people to adopt technological knowledge and culture from both China and India [11, 14].

Previous genome-wide studies found evidence of SA genetic ancestry in several present-day MSEA population [15, 16]. The SA ancestry in these populations ranged from 25% to 30% and dated to ∼500 to 750 ya in one study [15], while another study estimated SA admixture proportions ranging from ~4% to 12% and dates between ~450 to 1,600 [16]. Additional insights into SA ancestry in SEA could come from analyses of the paternally-inherited male-specific portions of the Y chromosome (MSY) and the maternally-inherited mitochondrial (mt) DNA genome. These markers are able to reconstruct different aspects of the genetic history than obtained from genome-wide data, especially sex-biased admixture, which frequently occurs in human populations [17–20]. Contrasting patterns of MSY and mtDNA variation were reported previously in some MSEA populations, especially the highlanders in Thailand and Vietnam [21–25], and shown to be influenced mainly by postmarital residence patterns (i.e., matrilocality vs. patrilocality), genetic drift, and admixture with lowlanders.

**Fig 1:**
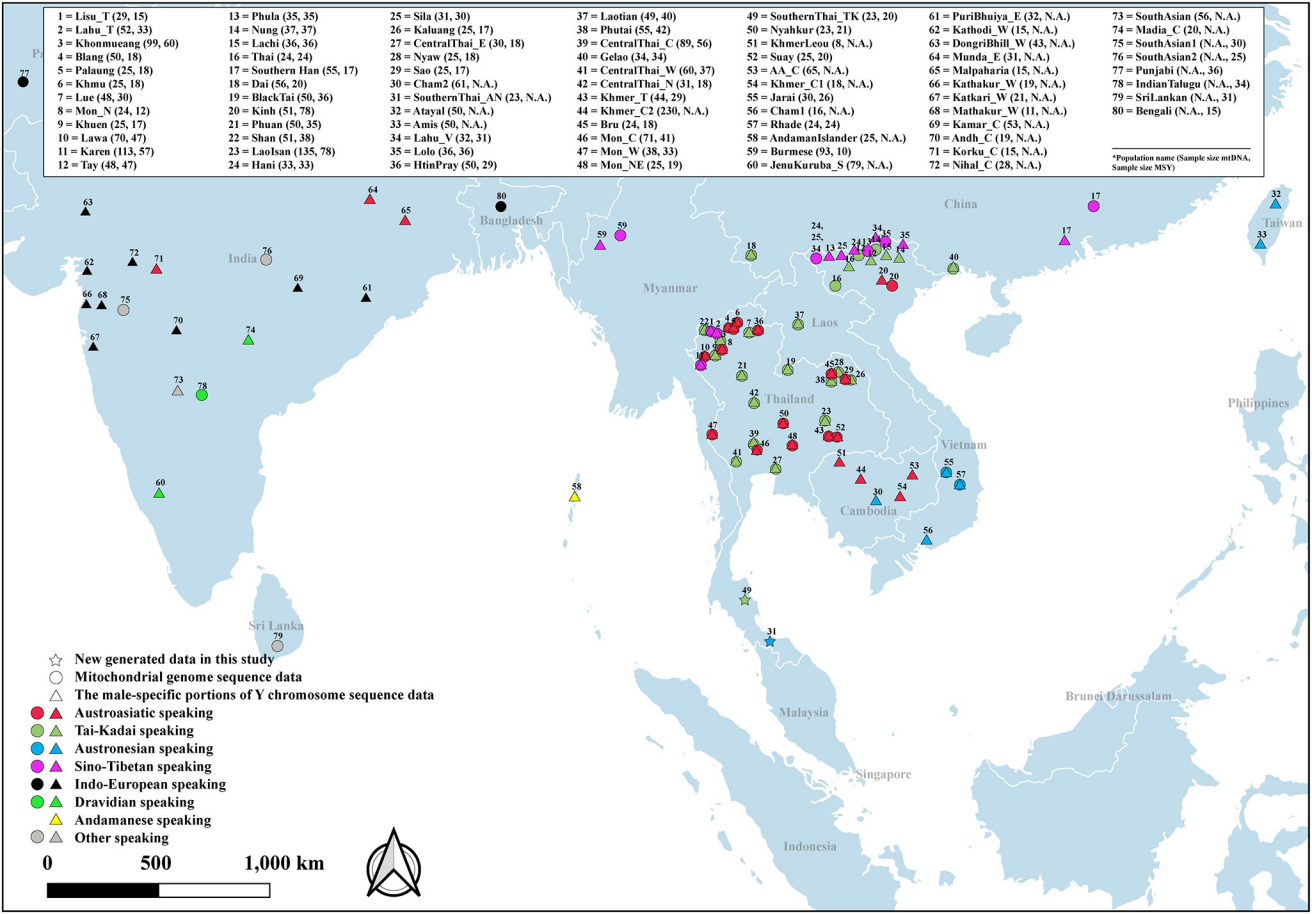
Map showing the locations of 88 populations from Southeast and South Asia that were analyzed for mtDNA (74 populations) and MSY (55 populations) variation. The map was generated using the Quantum GIS, QGIS Development Team (2023), QGIS Geographic Information System. Open Source Geospatial Foundation (https://www.qgis.org) and boundaries were adapted and modified from https://public.opendatasoft.com/explore/dataset/worldadministrative-boundaries/export/.

Previous MSY and mtDNA studies reported the presence of haplogroups frequent in SA in some Thai populations, e.g., mtDNA haplogroups W3a1b, M30 and M45 in central Thais and Mon [26, 27] and MSY haplogroups R1a1 in Khmer, J2a in Nyahkur and J and R in Mon [23]. However, the role of sex-biased admixture related to SA ancestry in MSEA has not been studied, and moreover only a few groups from Southern Thailand have been investigated for mtDNA and MSY variation [5, 28, 29]. Here, we generated new data, consisting of complete mtDNA genome sequences from TK- and AN-speaking southern Thai populations, and ∼2.3 mB of MSY sequences from the southern Thai_TK. We explored maternally and paternally genetic lineages and genetic relationships between southern Thais and other SA/SEA populations (Fig 1). We investigated SA genetic contribution in our newly-generated southern Thai populations as well as in published data from MSEA, and we find heterogeneity in SA admixture and sex-biased admixture in MSEA populations.

## Materials and methods

### Samples

The 46 genomic DNA samples (23 for southern Thai_TK and 23 for southern Thai_AN) were from previous studies [15, 30]. For southern Thai_TK, there are 20 males and 3 females but all 23 southern Thai_AN donors are females. Samples were recruited during November 2019. Sample donors were unrelated for at least two generations and provided buccal samples with written informed consent, after the goals of the study were explained and all questions answered. The rights and identity of all donors have been protected during the entire process of this research, according to guidelines and regulations based on the protocol on human subjects which was approved by the Khon Kaen University Ethic Committee (Protocol No. HE622223). Genomic DNAs were extracted using the Gentra Puregene Buccal Cell Kit (Qiagen, Germany) according to the manufacturer’s instructions.

### Sequencing

On December 2019, genomic libraries with double indices were prepared and enriched for mtDNA as described previously [31, 32]. The libraries were sequenced on an Illumina Hiseq 2500 and mtDNA consensus sequences obtained as described by previous study [19] with minor modifications. Bustard was used for Illumina standard base calling with 76 bp for read length. Sequences were then manually checked and manipulated with Bioedit (www.mbio.ncsu.edu/BioEdit/bioedit.html). The sequence alignment to the Reconstructed Sapiens Reference Sequence (RSRS) [33] was done by MAFFT 7.271 [34].

The same genomic libraries for 20 male samples of southernThai_TK were enriched for ∼2.3 mB of the MSY via in-solution hybridization-capture using a previously designed probe set [5, 23] and the Agilent Sure Select system (Agilent, CA). Sequencing was carried out on the Illumina HiSeq 2500 platform with paired-end reads of 125-bp length. Bustard was used for Illumina standard base calling, and leeHOM was used to trim Illumina adapters and merge completely overlapping paired sequences [35]. Then, deML was used to demultiplex the pooled sequencing data [36]. The alignment and post-processing pipeline of the sequencing data was carried out as previously described [5].

### Statistical analysis

The newly-generated 46 mtDNA sequences (S1 Table) were combined with 3,169 sequences from 72 populations from SA and SEA to obtain a broader picture of population relationships [24, 26, 37–47] for a total of 3,215 sequences belonging to 74 populations (S2 Table). Apart from these 74 populations, another published 31 sequences of haplogroup Q3 and 71 sequences of haplogroup E1a1a1 from SEA and Near Oceania were included in the median-joining network [48] of haplogroup Q3 [49–54] (S3 Table) and E1a1a1 [37, 55–57] (S4 Table). The network was constructed with Network 10.2 (www.fluxus-engineering.com) without pre- and post-processing steps, and all nucleotide position were weighted equally. For MSY, combining the 20 new sequences of southern Thai_TK from this study (S5 Table) with 1,674 sequences from previous studies [23–25, 58–60] brings the total to 1,694 sequences from 55 populations (S6 Table). For mtDNA, haplogroup assignment was performed by Haplogrep [61] with PhyloTree mtDNA tree Build 17 (http://www.phylotree.org) [62]. The mixture proportions of mtDNA samples and most likely detected mitochondrial haplogroups for each sample were estimated and identified by mixEMT software [63].

Using yHaplo [64], MSY haplogroups were assigned to the maximum depth possible given the phylogeny of the ISOGG Y-DNA Haplogroup Tree 2015 (http://www.isogg.org/) and the available genetic markers in our target region. Summary statistics of the genetic diversity within populations and the matrix of pairwise genetic distances (*Φ*_st_) were obtained with Arlequin 3.5.1.3 [65]. To visualize population relatedness, the R package (R Development Core Team 2016) [66] was used to carry out nonparametric MDS analysis (based on the *Φ*_st_ distance matrices for the MSY and mtDNA and using R function: isoMDS package: MASS) and to construct heat plots of the *Φ*_st_ distance matrix and the matrix of shared haplotypes (R function: ape, pegas, adegenet and ggplot2 packages). STATISTICA 13.0 (StatSoft, Inc., USA) was used to carry out a correspondence analysis (CA) based on MSY and mtDNA haplogroup frequencies.

Admix 2.0 was used to estimate admixture proportions [67, 68]. We used a simple admixture model consisting of two parental groups: East and Southeast Asian (ESEA) and South Asian (SA) (S2 and S6 Tables). Several analyses using 12 and 8 different parameters for mtDNA and MSY, respectively, i.e., admixture times (2000, 1000, 500, and 0 ya) and mutation rates (0, 1.67 x 10^−8^ and 2.67 x 10^−8^ for mtDNA and 0 and 8.71 x 10^−10^ for MSY) were performed (S7 Table) [69–71], with 10,000 bootstrap replicates.

To construct Bayesian skyline plots (BSP) per population, based on Bayesian Markov Chain Monte Carlo (MCMC) analyses, we used BEAST 1.8.4 [72]. BEAST input files were created with BEAUTi v1.8.2 after first running jModel test 2.1.7 in order to choose the most suitable model of sequence evolution [73]. For mtDNA, we executed BSP analyses per population with mutation rates of 1.708 × 10^−8^ and 9.883 × 10^−8^ for data partitioned between the coding and noncoding regions, respectively [69]. For the MSY, we used an MSY mutation rate of 8.71 × 10^−10^ substitutions/bp/year [71], and the BEAST input files were modified by an in-house script to add in the invariant sites found in our data set. Both strict and log normal relaxed clock models were run, with marginal likelihood estimation [74, 75]. After each BEAST run, the Bayes factor was computed from the log marginal likelihood of both models to choose the best-fitting BSP tree. Tracer 1.6 was used to generate the BSP plot from the BEAST results.

## Results

### Genetic diversity

We generated 46 complete mtDNA sequences from two populations, southernThai_TK and southernThai_AN, with mean coverage ranging from 788x to 4022x (overall average coverage 2346x) (S1 Table). There were no missing sites in any of the sequences, and an analysis for sample mixtures did not detect any evidence of multiple sequences in any sample. Compared to other populations in Thailand and elsewhere in Asia (S2 Table), high genetic diversity values for both populations were observed: haplotype diversity (*h*) = 1.00 for both southernThai_TK and southern Thai_AN, mean number of pairwise difference (MPD) = 36.9 for southernThai_TK and 34.7 for southern Thai_AN, Tajima’s *D* values = -2.18 (*P* < 0.01) for southernThai_TK and -1.99 (*P* < 0.01) for southern Thai_AN. Both the high genetic diversities and the significantly negative Tajima’s *D* values suggest recent maternal expansions in these two groups.

The mean coverage for the 20 new MSY sequences, for the SouthernThai_TK, ranges from 11.42x to 22.62x (overall average coverage 15.87x) (S5 Table). Genetic diversity values (*h* = 1.00, MPD = 98.8) indicate high genetic variation within this population compared to other Thai and SA/SEA populations (S6 Table). The Tajima’s *D* value of -2.04 (*P* < 0.01) also indicates a recent history of paternal population expansion.

### Genetic lineages

We assigned haplogroups as prevalent in SEA/EA (found at high frequency in SEA/EA and absent or nearly so in SA, e.g. B, F, M7, R*, and R9), prevalent in SA (e.g. M*(xM7), M8, M9 and M12), or other (found at low frequency in either or both regions, e.g. G, M12, and N*) (S1 Fig). Among a total of 22 mtDNA haplogroups detected in southernThai_TK, there were 39.13% SEA/EA prevalent haplogroups (B4a1a, F1a1a, F1f, M7b1a1 (+16192), M7c1a3, R9c1b1) and 43.48% prevalent in SA (M3c1a, M17, M21b, M21b2, M22a, M24a, M26, M38, M50, M77); other haplogroups, accounted for 17.39% (N, N8, M12a2, R11b1 and R11’B6) (S1 Fig, S8 Table). For the 18 haplogroups in southernThai_AN, SEA/EA prevalent haplogroups (B4c1b2a2, B4c2, B5a1a, F1a1a1, F1f, M7c1c3) had a frequency of 39.13%; SA prevalent haplogroups (M17c, M21a, M24a, M50a, M51b, M72a and M74b2) accounted for 34.78%, and other haplogroups (M12a, N9a6a, N22, Q3, E1a1a1) occurred with frequency 26.09% (S1 Fig, S8 Table).

Interestingly, there is one SouthernThai_AN individual with mtDNA haplogroup Q3, which has never been reported in MSEA populations. We retrieved mtDNA genomes of haplogroup Q3 from previous studies [49–54] and constructed a median-joining network to investigate the mtDNA relationships within Q3. The SouthernThai_AN Q3 sequence differed from all others but was closest to a sequence from East Timor (differing by 3 substitutions) and both sequences were more distantly related to a sequence from an individual belonging to the non-Austronesian-speaking Oro group from Papua New Guinea (S2 Fig). In addition, haplogroup E1a1a1, which has not been reported in any other Thai populations, was observed in SouthernThai_AN. The network constructed from mtDNA genomes of haplogroup E1a1a1 from previous studies [37, 55–57, 76] showed a star-like structure that reflected lineage expansion of this haplogroup. All E1a1a1 sequences branch off from the central haplotype, which is present in individuals from the Philippines, and carry only a few distinct mutations (1 to 2), with the exception of some haplotypes from Taiwan and one from Indonesia that are separated by four to five mutations from the central node (S3 Fig).

In other MSEA groups, SA prevalent haplogroups were in very high frequency (greater than 60%) in Burmese, three AN populations from Vietnam (Rhade, Jarai, Cham1) and Cambodian Khmer_C1, while all southern Thais, almost all Mon groups (except Mon_N), Nyahkur, Suay, and two populations from Cambodia (KhmerLeou and AA_C) exhibited moderately high levels of SA prevalent haplogroups (30–60%) (S4 Fig). All central Thai groups, Mon_N, Thai Khmer (Khmer_T), and Cambodian Khmer (Khmer_C2) had percentages of SA prevalent haplogroups ranging from 15–30%. The Correspondence Analysis (CA) based on haplogroup frequencies further illustrates these results, as the Burmese, Khmer_C1, Rhade, Jarai, and Cham1 are positioned closest to SA populations, and in general the MSEA AA-speaking populations were closer to SA populations than other SEA/EA language groups; the southern Thai_TK, differ notably from other TK groups in being closer to SA groups (S5 Fig).

There were 19 MSY haplogroups among the 20 newly generated sequences from the SouthernThai_TK; the SEA/EA prevalent haplogroups O1 and O2 (O1a1a1a1a, O1b1a1a, O1b1a2a1, O1b1a2b1, O2a1c1a1a1, O2a1c1a7, O2a2a1a2a1a, O2a2b1a1a3a, O2b1a) were the most frequent at 40% (S6 Fig, S5 Table). SA prevalent haplogroups, identified as belonging to H*, J*, L*, and R* (H1a1d2, L1a, R, R1a1a1b and R1a1a1b2a1b) had a frequency of 35%, and another 25% were other haplogroups (C, C2e2a1, Q1a2, K and N1c2) (S6 Fig, S9 Table). When other MSEA were included, in contrast to mtDNA, the CA analysis based on MSY haplogroup frequencies indicated that TK groups (including the SouthernThai_TK) were closer to the SA populations than AA groups (other than the Mon) (S7 Fig). This contrasting pattern was also observed in the AN-speaking Vietnamese groups. Specifically, SouthernThai_TK, the CentralThai_N, Mon_C and Mon_W had high percentages of SA prevalent haplogroups (greater than 30%) while Rhade, Jarai, Suay, Khmer, Mon_NE and Nyahkur exhibited frequencies lower than 15% (S8 Fig).

### Genetic relationships among populations

**Fig 2:**
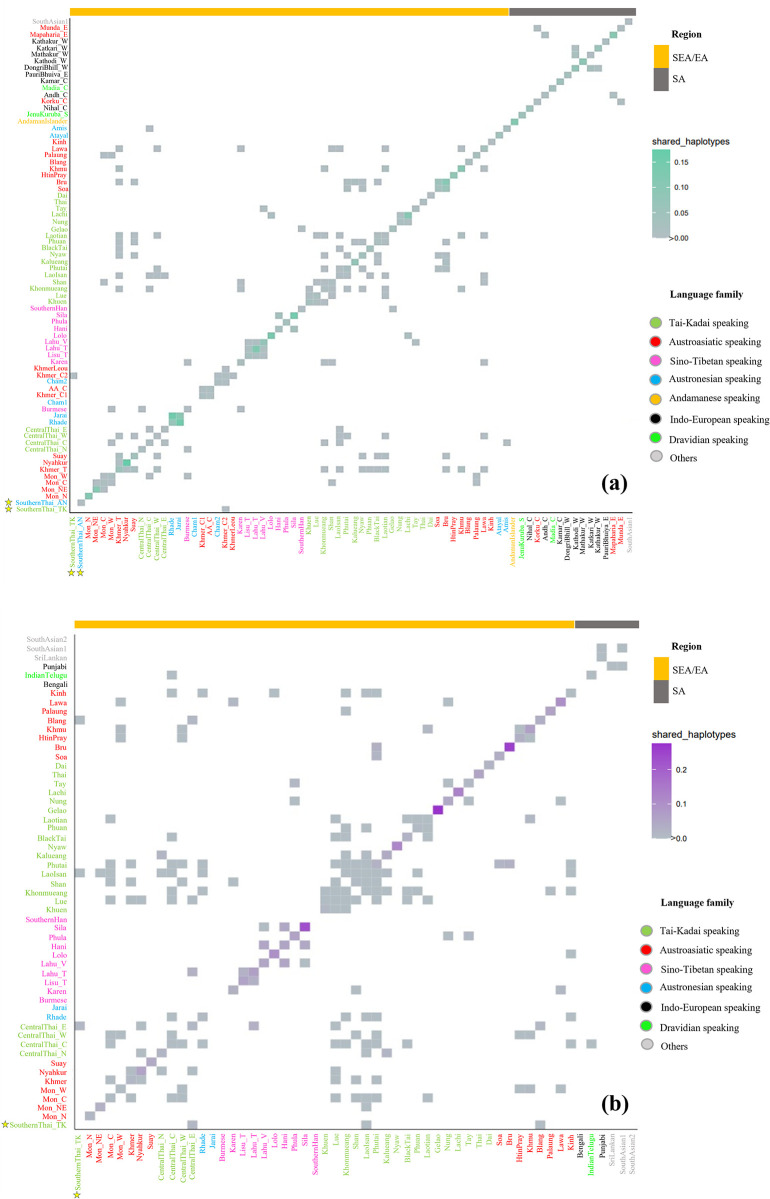
Frequency of shared mtDNA (a) and MSY (b) haplotypes within and between populations. The font color of the population name indicates language family and the colored bar at the top indicates geographic region; the heat plot shows the frequency of shared types according to the key on the right. The new populations analyzed in this study are indicated by stars.

Shared haplotypes within populations indicate smaller population size due to relatedness among individuals, whereas shared haplotypes between populations could reflect recent shared ancestry or contact. We observe some shared mtDNA haplotypes within the southernThai_AN but not in southernThai_TK, but some sharing between southernThai_TK and Khmer from Cambodia (Khmer_C2) (Fig 2a). No SA group shared mtDNA haplotypes with SEA/EA groups (Fig 2a).

**Fig 3:**
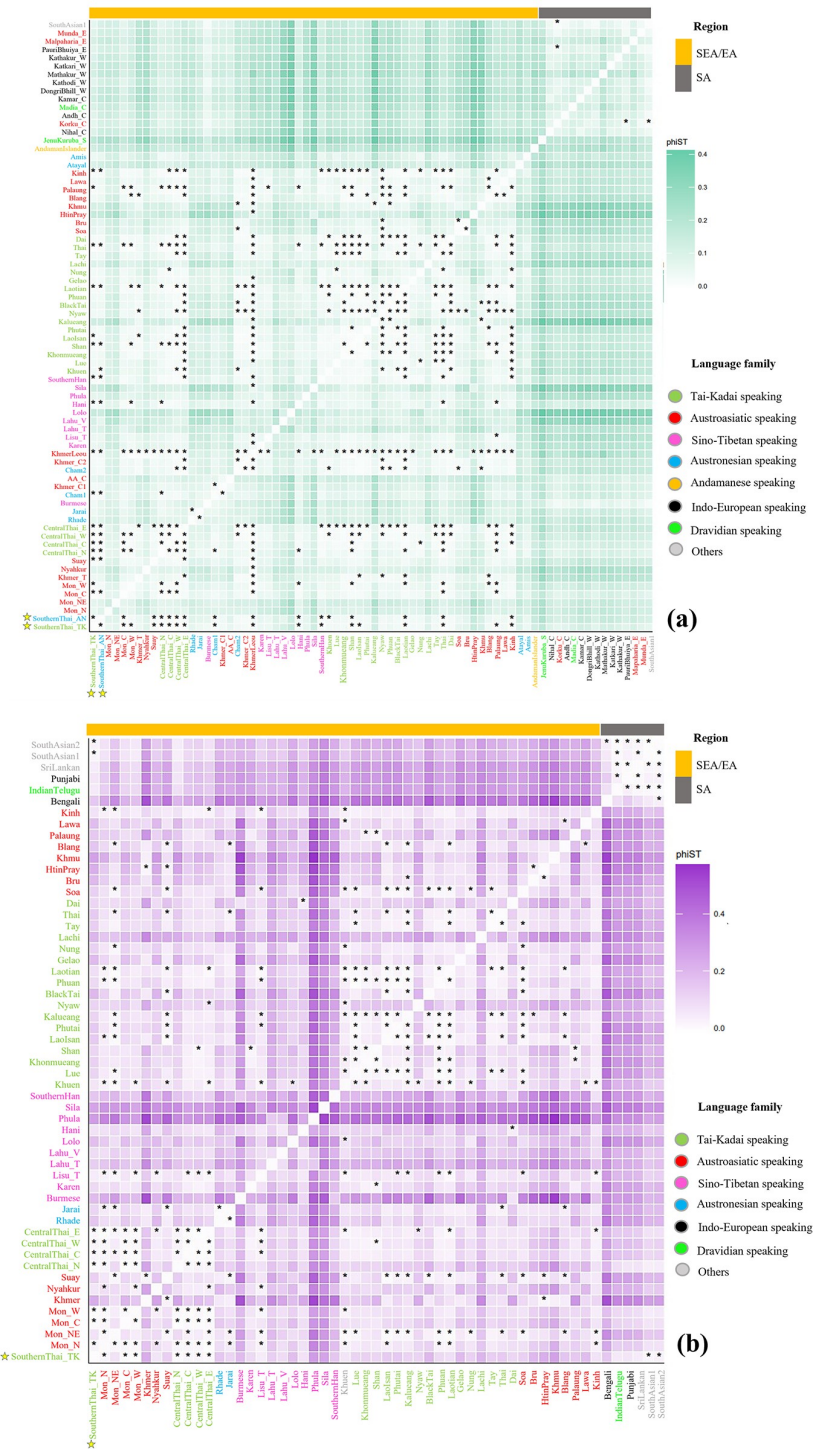
Heat plot of *Φ*_st_ values based on mtDNA (a) and MSY (b) haplotypes. The “*”symbol indicates *Φ*_st_ values that are not significantly different from zero (*P* > 0.05). See legend of Fig 2 for other details.

Genetic relationships between populations can be inferred by measuring genetic distances. Pairwise *Φ*_st_ genetic distances based on mtDNA showed no significant differences between the two southern Thai populations, and between these and several other populations, such as Mon_C, all central Thais and Shan from Thailand, Cham1, Hani, Thai and Kinh from Vietnam, Cambodian KhmerLeou, southern Chinese Han, and Laotian populations, as shown in Fig 3a. In general, SA populations exhibited smaller genetic distances among them and larger genetic distances to most populations from SEA. However, southern Thais, central Thais, Mon, Burmese, Cambodian, and AN-speaking Rhade, Jarai, and Cham from Vietnam showed smaller distances to SA groups than to other SEA populations (Fig 3a).

**Fig 4:**
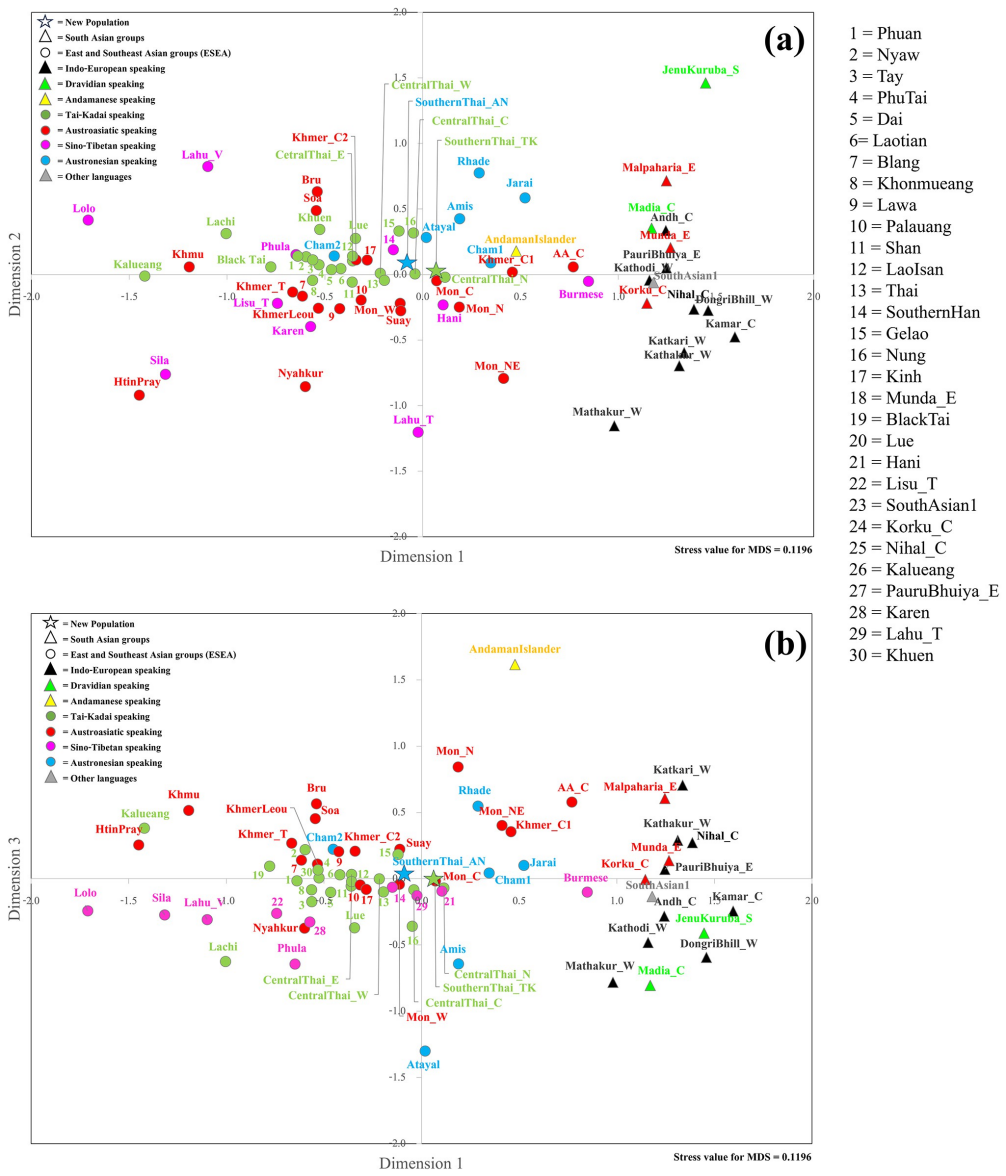
The three-dimensional MDS plot based on the *Φ*_st_ distance matrix of 74 populations for mtDNA. The stress value is 0.1196.

Interestingly, AN-speaking Amis and Atayal were genetically different from AN-speaking groups from Vietnam and also from other SEA populations (Fig 3a). A multidimensional scaling (MDS) plot was employed to visualize further genetic relationships based on the *Φ*_st_ distance matrix. The MDS plots based on three dimensions indicates genetic separation between SA and SEA/EA populations with some SEA populations, i.e., southern Thais, some central Thais and Mon groups in Thailand, Burmese, Cambodians and Cham1, Jarai and Rhade from Vietnam between these two clusters, suggesting an admixed genetic structure (Fig 4a and 4b).

**Fig 5:**
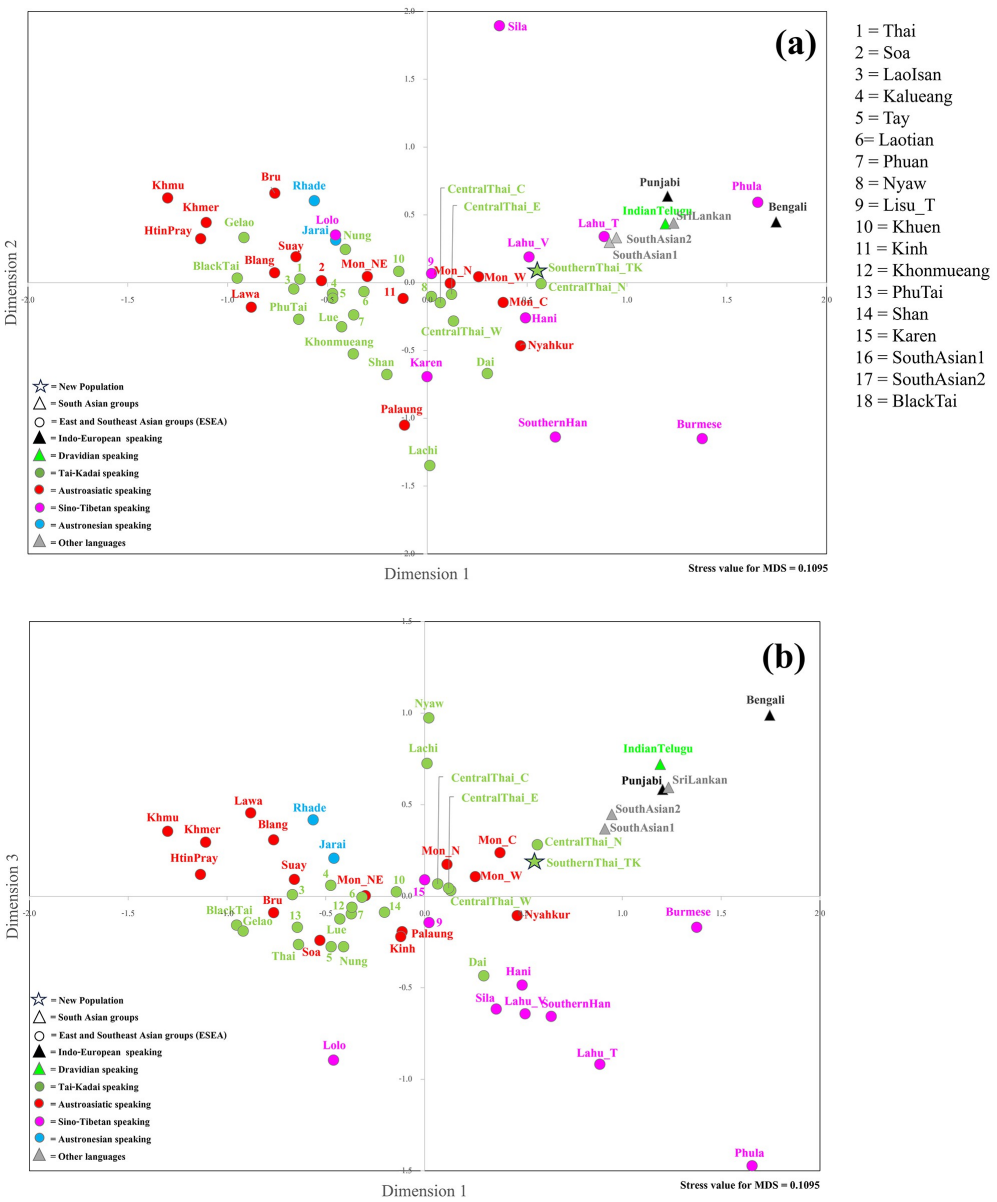
The three-dimensional MDS plot based on the *Φ*st distance matrix of 55 populations for MSY sequences. The stress value is 0.1095.

For the MSY, southernThai_TK shared haplotypes with centralThai_E, Lao Isan and AA-speaking Blang from northern Thailand (Fig 2b). In general, we observed more shared haplotypes between AA- and TK-speaking groups in Thailand than between any other groups. SA populations shared more haplotypes among themselves than with SEA groups; only IndianTelugu shared haplotypes with centralThai_C. The heat plot of *Φ*_*st*_ genetic distances also supports non-significant differences between southernThai_TK and all central Thai populations (Fig 3b). In contrast to mtDNA, the southernThai_TK also exhibit genetic similarity with almost all Mon populations, except Northeastern Thai Mon (Mon_NE), and two South Asian populations. The SA populations showed genetic similarities among themselves and all of them differed significantly from SEA populations (except the two SA populations with the southernThai_TK, as noted above). The MDS plots based on *Φ*_st_ values show genetic heterogeneity of SEA Sino-Tibetan-speaking populations; they are also differentiated from a cloud of SA populations and a cloud of other SEA populations. The southernThai_TK, some Mon and central Thai populations, Burmese, Phula, Vietnamese Lahu and Thai Lahu are positioned closer to the SA cloud than are other SEA populations (Fig 5a and 5b). In contrast to the mtDNA results, Jarai and Rhade had more affinity with SEA AA-speaking groups than with SA groups. The overall results from the genetic distance analyses indicate a contrasting pattern between maternal and paternal lineages in some SEA populations. For example, SouthernThai_TK, CentralThai_N, Nyahkur, and Mon_C tend to show paternal genetic relationships closer to SA than do the maternal lineages in these groups (Fig 5), while Rhade, Jarai, and Mon_NE showed the opposite pattern (Fig 4).

### Genetic contribution of South Asian to Southeast Asian populations

The results based on genetic lineages and genetic distance analyses indicate that southern Thais and several MSEA populations exhibit genetic relatedness to both SEA and SA populations, with some exhibiting contrasting mtDNA vs. MSY patterns, e.g. Mon, Nyahkur, Central Thai, Khmer, Burmese, Cham, Jarai and Rhade. All results support SA genetic influences in those groups that are consistent with genome-wide studies [15, 16]. Assuming that these results are indeed indicative of admixture, we then used a model-based approach to estimate the proportion of SA and SEA ancestry in these admixed groups. We used a simple admixture model consisting of two parental groups, East and Southeast Asian (ESEA) and South Asian (SA) (S2 and S6 Tables), using the coalescent-based method implemented in Admix 2.0 [67, 68]. The ESEA parental groups were chosen based on the SA admixture results from previous studies, which reported no significant SA-related components [15, 16] and based on haplogroup distributions ESEA surrogates harbor very few SA lineages and SA source has very few (if any) ESEA lineages (S4 and S8 Figs). According to historical records and archaeological evidence as well as recent ancient DNA studies [77], the interaction between SA and MSEA started at most 2 kya; we therefore considered admixture times starting 2 kya. We performed several analyses using different parameters, i.e., admixture times (2000, 1000, 500, and 0 ya) and mutation rates (0, 1.67 x 10^−8^ and 2.67 x 10^−8^ for mtDNA and 0 and 8.71 x 10^−10^ for MSY) (S7 Table) [69–71]. Because admixture results were consistent in all analyses (S10 and S11 Tables), we consider admixture results at 2,000 years since the admixture event occurred with mutation rates of 1.67 x 10^−8^ for mtDNA and 8.71 x 10^−10^ for the MSY.

**Fig 6:**
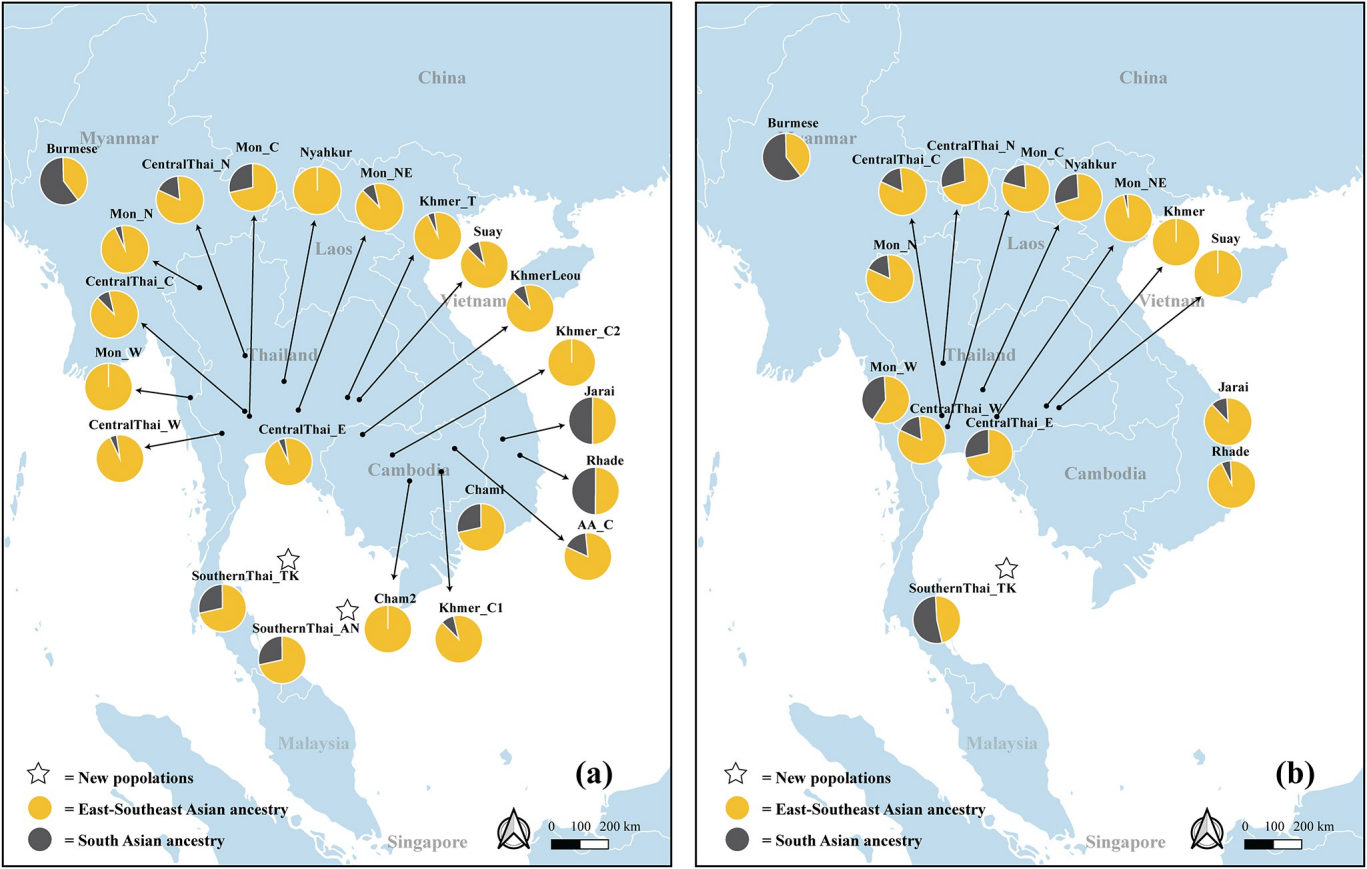
Pie charts of South Asian ancestry in Southeast Asian populations. (a) mtDNA, 22 populations; (b) MSY, 15 populations. The map was generated using the Quantum GIS, QGIS Development Team (2023), QGIS Geographic Information System. Open Source Geospatial Foundation (https://www.qgis.org) and was not taken from another source. and boundaries were adapted and modified from https://public.opendatasoft.com/explore/dataset/worldadministrative-boundaries/export/.

The maternal contribution from SA to SEA was highest in Burmese (61.4%), Rhade (50.0%), and Jarai (49.3%), while levels of SA ancestry in SouthernThai_TK, SouthernThai_AN, Mon_C, CentralThai_N, AA_C and Vietnamese Cham1 ranged from ~15–30%. Other populations showed SA contributions lower than 15%, and no SA contribution was detected in Khmer_C2, Cham2, Nyahkur and Mon_W (Fig 6a). For the MSY (Fig 6b), Burmese and SouthernThai_TK had the highest estimated SA ancestry (59.4% and 54.32%) while Mon_W had 38.83% SA-related ancestry. Mon_N, Mon_C, Nyahkur, and all Central Thai populations exhibited SA admixture ranging from ~15% to ~30%. The Jarai, Rhade and Mon_NE had low levels of SA admixture (~2–10%) while Suay and Khmer showed no detectable SA admixture. As discussed in more detail below, we thus find contrasting patterns of mtDNA vs MSY SA admixture in practically all populations, except for Burmese, who have the highest level of SA ancestry for both markers.

### The effective population size change over time

**Fig 7:**
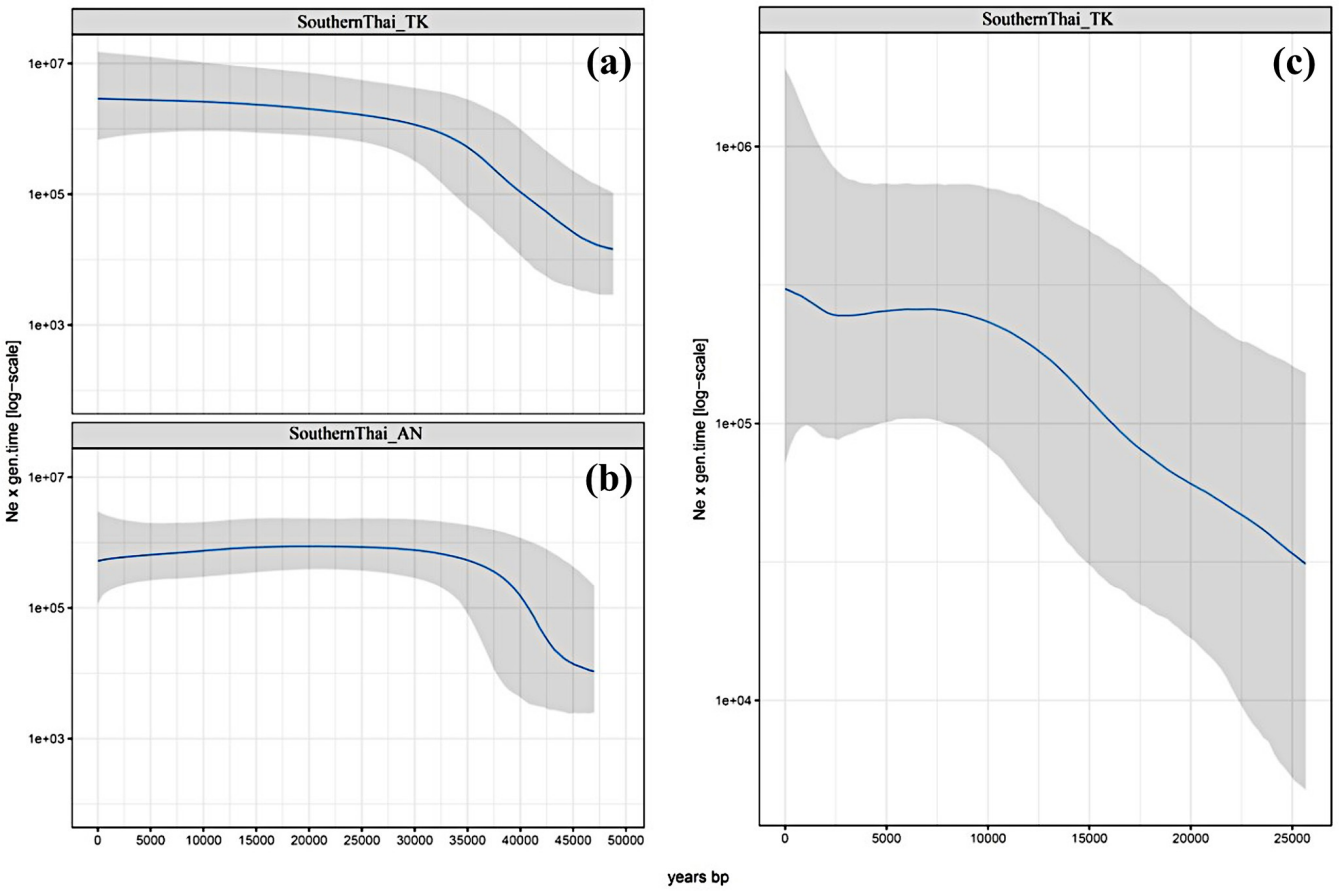
The BSPs based on mtDNA for the SouthernThai_TK (a) and SouthernThai_AN (b), and based on MSY for the SouthernThai_TK (c). Blue lines are the median estimated effective population size (y axis) through time from the present in years (x axis). The 95% highest posterior density limits are indicated by light grey shading.

The Bayesian skyline plots (BSPs) of population size change (*N*_*e*_) over time based on mtDNA exhibit similar patterns for both Southern Thai populations newly sequenced here: *N*_*e*_ increased from 45 kya to 30 kya and then was stable until the present day (Fig 7a and 7b). For the MSY, the *N*_*e*_ of SouthernThai_TK increased from 25 kya until ~7.5 kya, and then remained constant until a slight increase is observed at ~2.5 kya (Fig 7c). The pattern of mtDNA *N*_*e*_ of Southern Thai groups is similar to other Central Thai populations [27], whereas the MSY *N*_*e*_ of southernThai_TK was unique in that no reduction in population size was detected throughout their demographic history, while for all TK groups a population size decline was observed between ~5–2.5 kya [23]. However, the size increase in SouthernThai_TK ~3–2 kya, is characteristic of other TK populations [23].

## Discussion

Previous studies based on autosomal markers revealed unique aspects of southern Thai populations compared to other Thai populations; in particular, the presence of South Asian–associated ancestry [15]. Although sex-biased admixture of the human populations can be revealed by genomic data based on comparing X chromosomal and autosomal variation [78–80], there are some limitations of using genome-wide data to infer sex-biased admixture, e.g. when there is an extremely low ancestry proportion, low number of X chromosomal SNPs, and potential differences in ascertainment bias between the X chromosomal and autosomal SNPs on a genotyping array [78]. Studies of mtDNA and MSY variation provide a direct assessment of sex-biased admixture, and moreover these uniparental markers can also provide additional information on specific or new genetic lineages. Here, we analyze new complete mtDNA genomes and ∼2.3 mB of the MSY in two southern Thai populations to reveal genetic lineages, demographic changes and their genetic structure based on sequencing data, and we additionally analyze sex-biased admixture involving South Asian ancestry in several other MSEA populations, including two southern Thai groups, that have evidence of South Asian ancestry in genome-wide data [15, 16].

While all of the MSY lineages found in our study have been found previously in MSEA, we did find two novel mtDNA haplogroups, E1a1a1 and Q3, in SouthernThai_AN; these two haplogroups have not been reported in MSEA. Haplogroup Q3 is particularly interesting, as the origin of haplogroup Q and sublineages has been placed in Northern Sahul (highland and coastal New Guinea and Near Oceania) [54], and it has not been reported previously from MSEA or western ISEA. The network of haplogroup Q3 sequences showed that the most closely related sequence to the southernThai_AN Q3 sequence comes from East Timor (S2 Fig). A recent study of ancient DNA from Wallacea found evidence for contact between MSEA and the Nusa Tenggara Islands of southern Wallacea (which include East Timor) [81]; while it is tempting to speculate that the Q3 sequence may also reflect such contact, it could also reflect other episodes of contact, as the Timor area was suggested to be a secondary pathway that modern humans used to travel between Sundaland and Sahul after initial colonization [82].

Haplogroup E1a1a1 that is abundant in Taiwan and ISEA [37, 83]. The network of haplogroup E1a1a1 sequences showed a star-like structure (S3 Fig), suggesting lineage expansion of E1a1a1 in the ISEA that is probably associated with the spread of Austronesian languages. In addition, some of the SEA/EA mtDNA haplogroups in Southern Thais, e.g., B4a1a and M7c1c3 are present elsewhere mainly in aboriginal Taiwanese and ISEA populations [84]. The sharing of these recent sublineages indicates contact between MSEA and ISEA, which has not been observed previously from Thai/Lao mtDNA data [23, 24, 26]. However, the AN-speaking southern Thai from this study exhibited closer genetic relatedness to MSEA populations than to the AN-speaking Amis and Atayal from Taiwan (Fig 3a). While the AN-speaking Cham populations from Vietnam and Cambodia are similar to SouthernThai_AN in their relationships with other populations (Fig 3a), the AN-speaking Jarai and Rhade from Vietnam exhibit a distinct maternal genetic structure. The ancestors of AN and TK groups were thought to originate in Southeast China ~6–5 kya and then they split. TK ancestors moved southward to MSEA whereas AN ancestors entered Taiwan and spread out of Taiwan to ISEA ~4–3 kya, thus Taiwan is likely the original home for AN groups [37, 85, 86]. Previous mtDNA studies of AN speaking Cham, Jarai and Rhade suggested that cultural diffusion played an important role in shaping the genetic structure of those groups [25, 38, 39], while a genome-wide study supported both migration and cultural diffusion [87]. Here, we suggest that the Southern Thai_AN language was introduced largely by cultural diffusion, but the mtDNA haplogroup profile does indicate some links with AN-speaking people from ISEA or alternatively, the southernThai_AN might reflect a migration of AN speakers to southern Thailand, followed by extensive admixture with local populations.

Interactions between SA and MSEA probably occurred since the late Bronze Age to Iron Age and influenced the formation of early states or civilizations, e.g. Pyu, Funan, Champa, Dvaravati, and Langkasuka [11, 14]; a recent ancient DNA study documented a high genetic contribution (~40–50%) from South Asia in prehistoric people who lived during the early period of Funan, one of the earliest states in MSEA in present-day Cambodia [77]. The later expansion of Indian culture in SEA might further be continued through the historical period by the activity of additional Indian immigrants [88], which could affect the overall demographic structure of MSEA populations. Previous genetic dating of the SA admixture in most present-day MSEA populations ranged during the historical period from ~400 to ~800 ya [15, 16] with the older dates in Nyahkur, Khmer, Jarai and Rhade [16]; these results support significant historical South Asian interaction in MSEA. Although both autosomal DNA studies of ancient samples and present-day people provided new information on the level of SA admixture in MSEA population and admixture dates that started since the Iron Age through the historical period, sex-specific demographic histories are more difficult to explore with autosomal SNPs.

Several analyses based on haplogroup frequency (S4–S8 Figs), and genetic distance (Figs 3–5) indicated the SA ancestry in several Thai and MSEA populations for both mtDNA and MSY. Admixture analysis based on model-based estimates also supported SA proportions in these MSEA populations (S10 and S11 Tables) with heterogeneous patterns of sex-biased admixtures that were detected among groups (Fig 6). We find less sex-biased admixture in Burmese and they have high SA proportions (~60%) for both mtDNA and MSY (Fig 6). Myanmar shares a border with India to the West, which would allow multiple migrations and promote substantial gene flow. The other MSEA groups exhibited stronger sex-biased admixtures. In general, based on mtDNA, almost all of the AA- and AN- speaking groups are genetically closer to SA than TK groups, and the SouthernThai_TK was notably different from CentralThai groups in being closer to SA groups (Fig 4, S4 and S5 Figs). The AA-speaking Mon_W is an exception since they have no mtDNA SA ancestry but elevated MSY SA ancestry (~40%) (S10 Table). SA admixture in Mon would have resulted from Mon-Burmese interactions. Mon, Burmese and Karen have been reported to share a mutation in the G6PD gene [89], which supports their close genetic affinities.

However, there are heterogenous sex-biased admixture patterns among Mon populations in Thailand. Historically, multiple (at least nine) migrations of the Mon groups from Myanmar to Thailand occurred during the 16^th^ to 19^th^ centuries A.D. [90, 91]; different spatial and temporal migrations could promote various interactions between either Mon and TK groups or new Mon and old settled Mon groups, which then led to heterogenous sex-biased admixture patterns and contrasting maternal and paternal genetic variation [17, 24, 25].

Interestingly, the AN-speaking groups in Vietnam (e.g. Jarai and Rhade) also exhibited a strong sex-biased SA admixture pattern. The Jarai and Rhade showed almost 50% SA maternal ancestry, but less than 10% SA paternal ancestry (Fig 6 and S10 and S11 Tables). Jarai and Rhade live in the highland area and are matrilocal, but some sources indicate a previous connection with Cham, the neighboring lowland population in Vietnam [92]. This connection is also supported by the results from a recent genome-wide data analysis [87]. There is evidence that the ancestors of Vietnamese AN groups might have had matrilineal societies [93] and might have interacted with each other and also with other MSEA groups, e.g., Khmer, Jarai and Rhade probably received SA components from other lowland populations and subsequently other factors, e.g., residence pattern (matrilocal in this case) could have influenced patterns of genetic variation.

In conclusion, we have expanded the study of high-resolution mtDNA and MSY sequences in MSEA with new data from Southern Thai populations, although a limitation of our study is that the sample sizes are relatively small. However, the overall agreement with previous studies suggests that our results are indeed representative of Southern Thais. We explored additional insights into the genetic histories of MSEA populations; the mtDNA haplogroups E1a1a1, Q3, B4a1a and M7c1c3 that were found in Southern Thai populations reflect MSEA and ISEA connections. Both southern Thai populations exhibited close genetic relatedness with AA-speaking Mon and Central Thais, and SA ancestry was one of the shared characteristics. Consistent with previous autosomal DNA studies, evidence of SA ancestry was found in some Thai and other MSEA groups based on analyses of specific lineages, haplogroup frequencies, and genetic distance analyses. The model-based estimates of admixture proportions confirm these results for both mtDNA and MSY. However, patterns of sex-biased admixture vary greatly among groups. Burmese show little sex bias and have elevated SA ancestries for both mtDNA and MSY and this probably reflects their close position to India. The other groups exhibit strong sex bias–for example, Jarai and Rhade in Vietnam, which exhibit more SA mtDNA than MSY ancestry, probably influenced by population interactions and cultural practices. In sum, new mtDNA genetic lineages (e.g., Q3 and E1a1a1) and evidence for sex-biased South Asian admixture in several MSEA populations from our study support the usefulness of high-resolution uni-parental markers to reconstruct aspects of the genetic history of populations that have not been previously revealed by autosomal markers.

## Supporting information

S1 Fig Bar plot of percentages of each major mtDNA haplogroup in four groups of populations: SouthernThai_TK, SouthernThai_AN, Southeast Asian/East Asian (SEA/EA) and South Asian (SA). Data from 56 SEA/EA populations, and 16 SA populations.
(TIF)

S2 Fig Median-joining network based on 32 mtDNA genomes of haplogroup Q3 from Southern Thailand, East Timor, and Papua New Guinea. Number of substitutions are shown on the lines connecting the nodes.
(TIF)

S3 Fig Median-joining network based on 72 mtDNA genomes of haplogroup E1a1a1 from Southern Thailand, Taiwan, the Philippines, Indonesia and Saudi Arabia. Number of substitutions are shown on the lines connecting the nodes.
(TIF)

S4 Fig Bar plot of percentages of three categories of mtDNA haplogroups in 74 populations: Southeast Asian/East Asian (SEA/EA) prevalent, South Asian (SA) prevalent and other. (TIF)

S5 Fig Correspondence analysis (CA) plot based on mtDNA haplogroup frequencies in 74 populations. (PDF)

S6 Fig Bar plot of percentages of each major MSY haplogroup in three groups of populations: SouthernThai_TK, Southeast Asian/East Asian (SEA/EA) and South Asian (SA). Data from 55 SEA/EA populations and 16 SA populations.
(TIF)

S7 Fig Correspondence analysis (CA) plot based on MSY haplogroup frequency of 55 populations. (PDF)

S8 Fig Bar plot of percentages of three categories of MSY haplogroups: Southeast Asian/East Asian (SEA/EA) prevalent, South Asian (SA) prevalent, and other haplogroups, in 55 populations. (TIF)

S1 Table Sequencing coverage and mtDNA haplogroup of each individual. (XLSX)

S2 Table General information on the studied populations and mtDNA genetic diversity values. (XLSX)

S3 Table General information on additional mtDNA sequences of haplogroup Q3, used in the network analysis. (XLSX)

S4 Table General information on additional mtDNA sequences of haplogroup E1a1a1, used in the network analysis. (XLSX)

S5 Table Sequencing coverage and MSY haplogroup of each SouthernThai_TK individual. (XLSX)

S6 Table General information on the studied populations and MSY genetic diversity values. (XLSX)

S7 Table Parameters in the admixture analysis. (XLSX)

S8 Table MtDNA haplogroup frequencies. (XLSX)

S9 Table MSY haplogroup frequencies. (XLSX)

S10 Table Estimated admixture proportions in admixed populations based on mtDNA sequences. (XLSX)

S11 Table Estimated admixture proportions in admixed populations based on MSY sequences. (XLSX)
