# Supplementary figures and images for: South Asian maternal and paternal lineages in southern Thailand and the role of sex-biased admixture
Source: PLoS One. 2023 Sep 14;18(9):e0291547. doi: 10.1371/journal.pone.0291547 (PMC10501589; doi:10.1371/journal.pone.0291547)

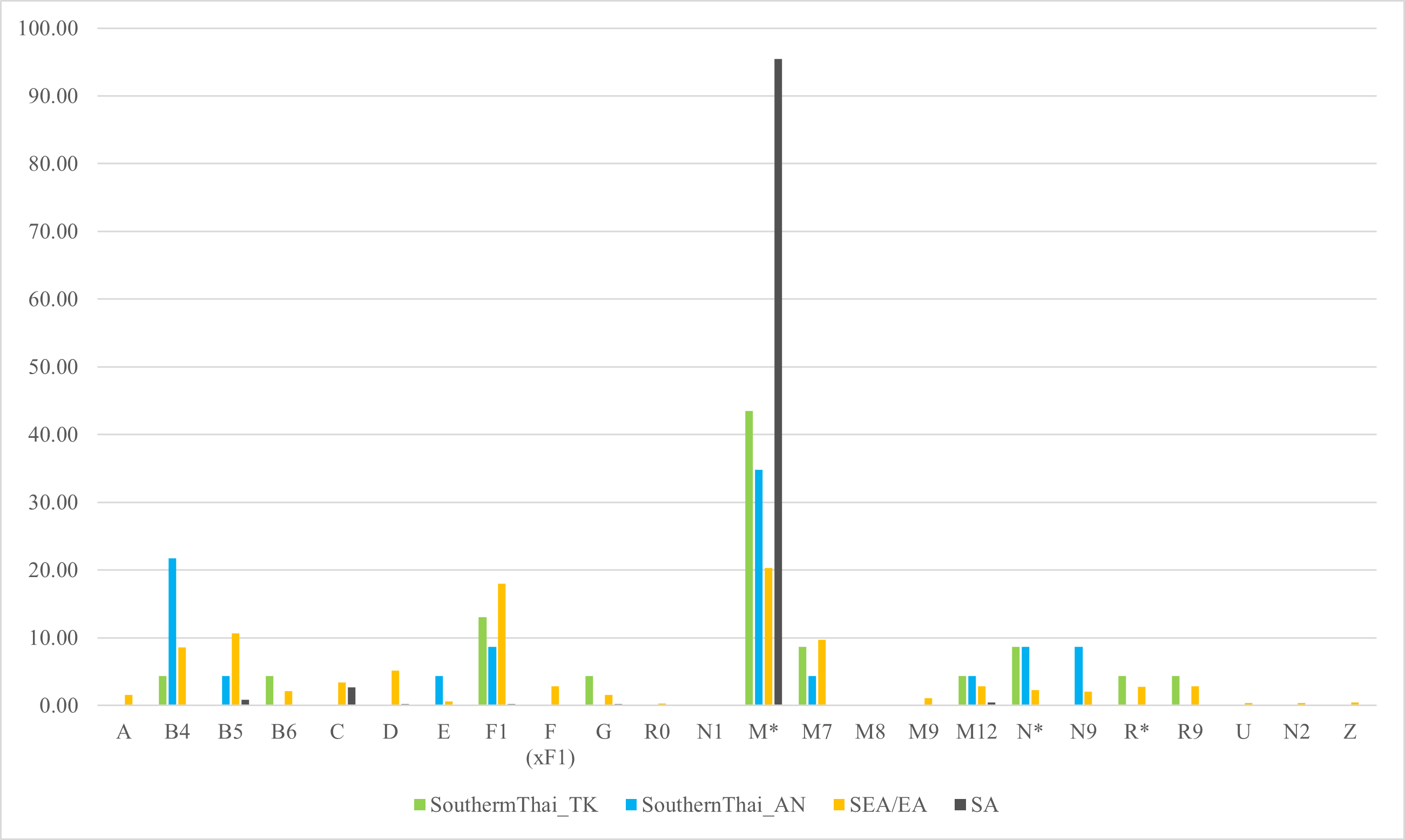

Supplement: S1 Fig — Data from 56 SEA/EA populations, and 16 SA populations. (TIF) [file pone.0291547.s001.tif]

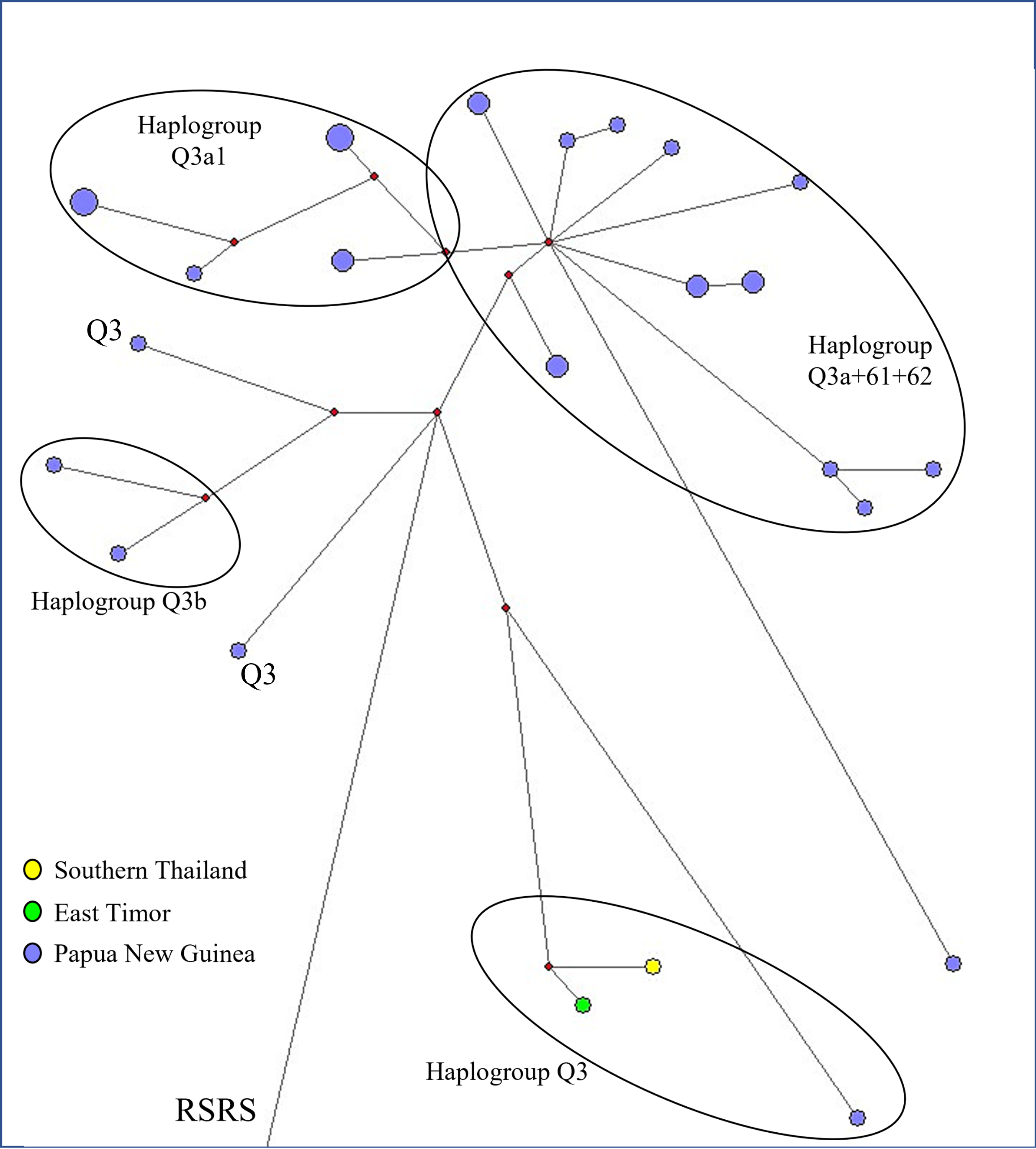

Supplement: S2 Fig — Number of substitutions are shown on the lines connecting the nodes. (TIF) [file pone.0291547.s002.tif]

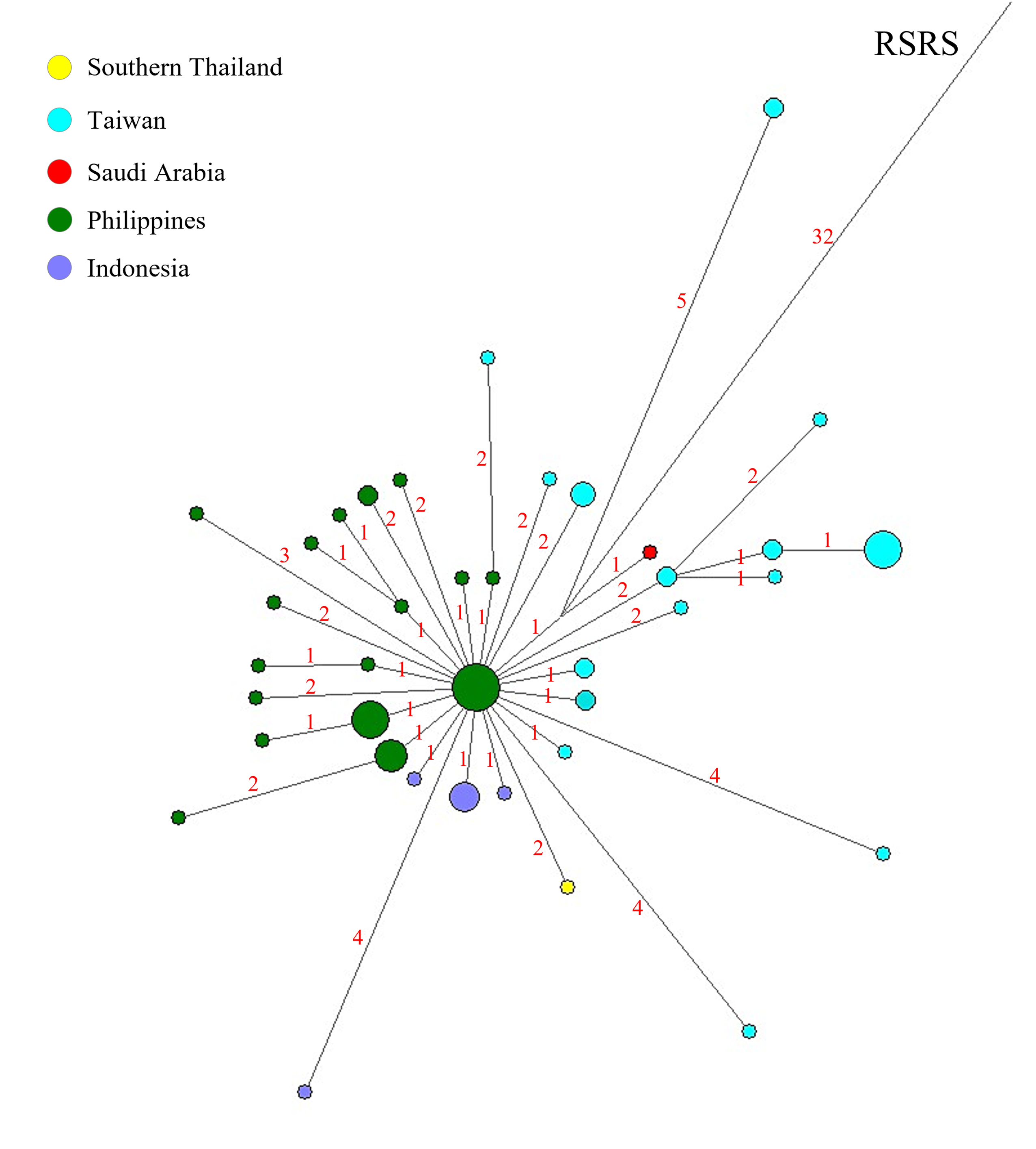

Supplement: S3 Fig — Number of substitutions are shown on the lines connecting the nodes. (TIF) [file pone.0291547.s003.tif]

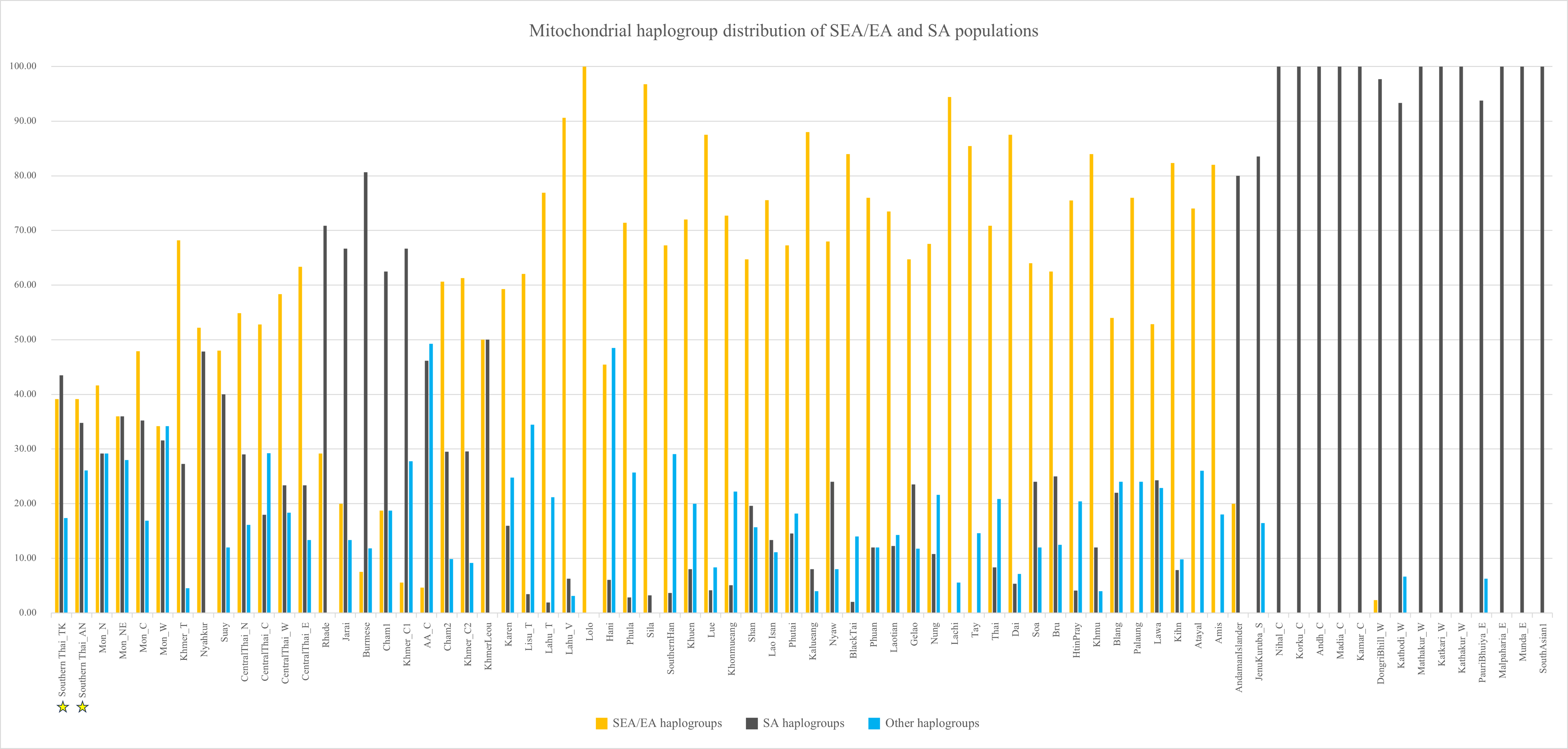

Supplement: S4 Fig — (TIF) [file pone.0291547.s004.tif]

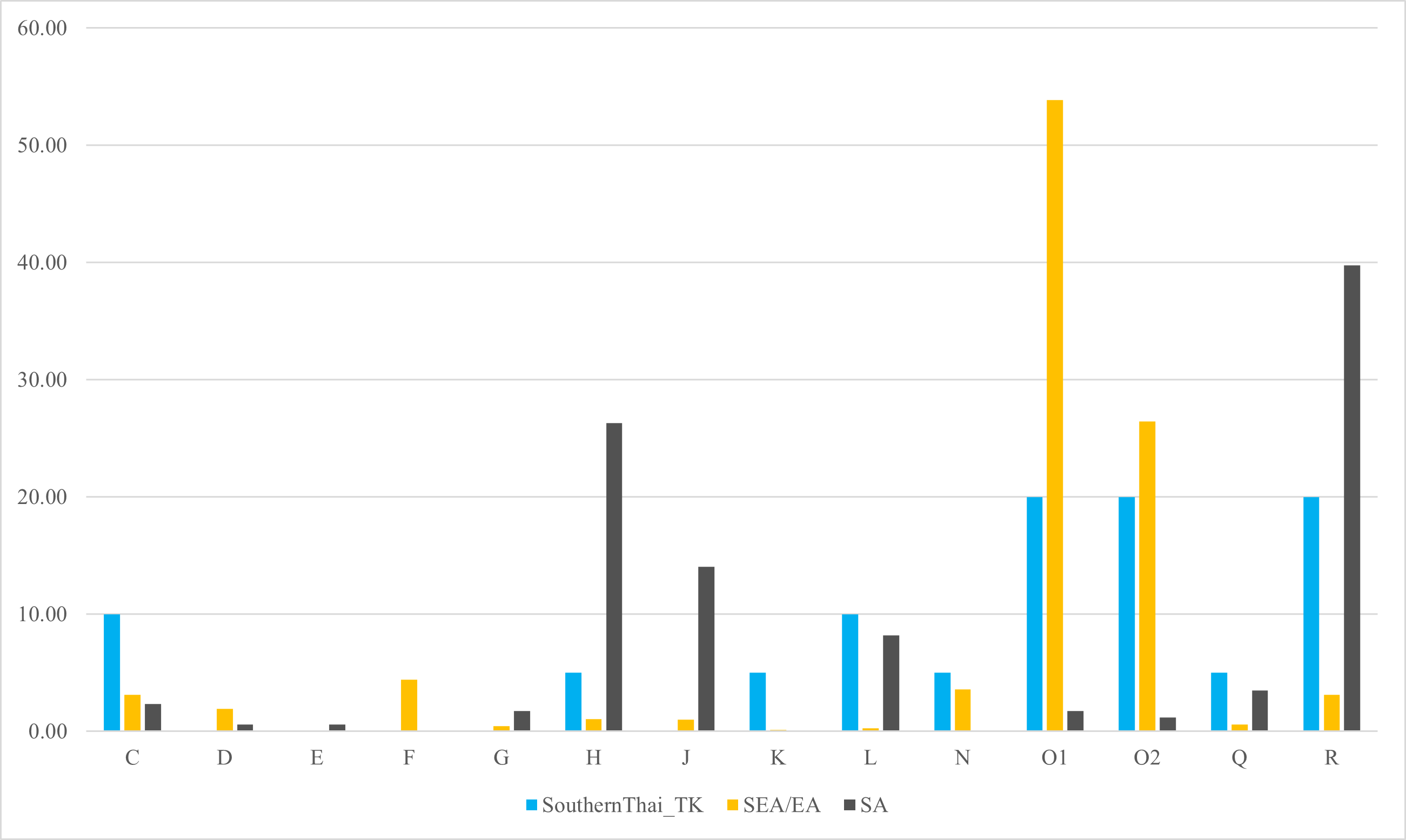

Supplement: S6 Fig — Data from 55 SEA/EA populations and 16 SA populations. (TIF) [file pone.0291547.s006.tif]

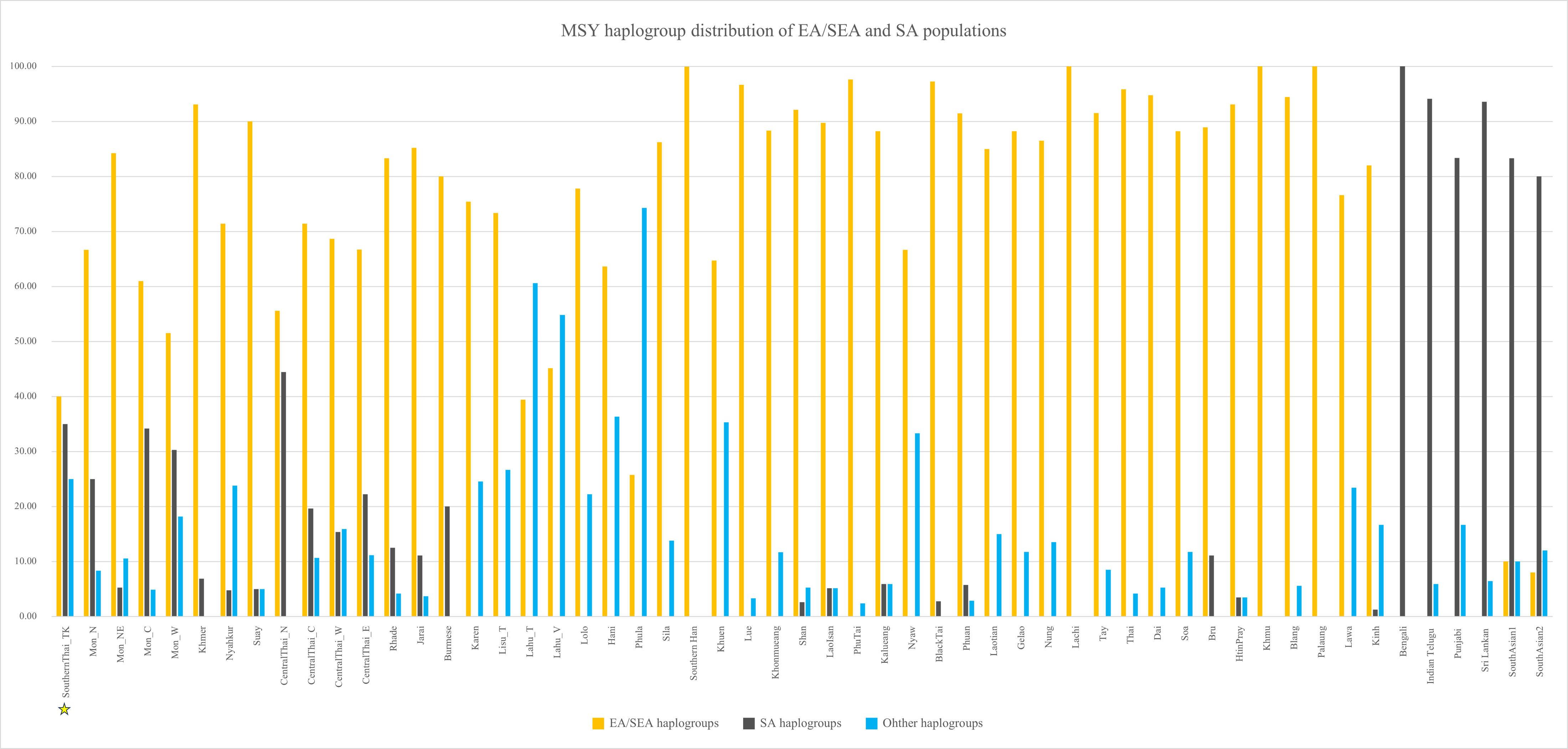

Supplement: S8 Fig — (TIF) [file pone.0291547.s008.tif]
