# Supplementary material for: South Asian maternal and paternal lineages in southern Thailand and the role of sex-biased admixture
Source: PLoS One. 2023 Sep 14;18(9):e0291547. doi: 10.1371/journal.pone.0291547 (PMC10501589; doi:10.1371/journal.pone.0291547)
Supplement: S7 Fig — (PDF) [file pone.0291547.s007.pdf]

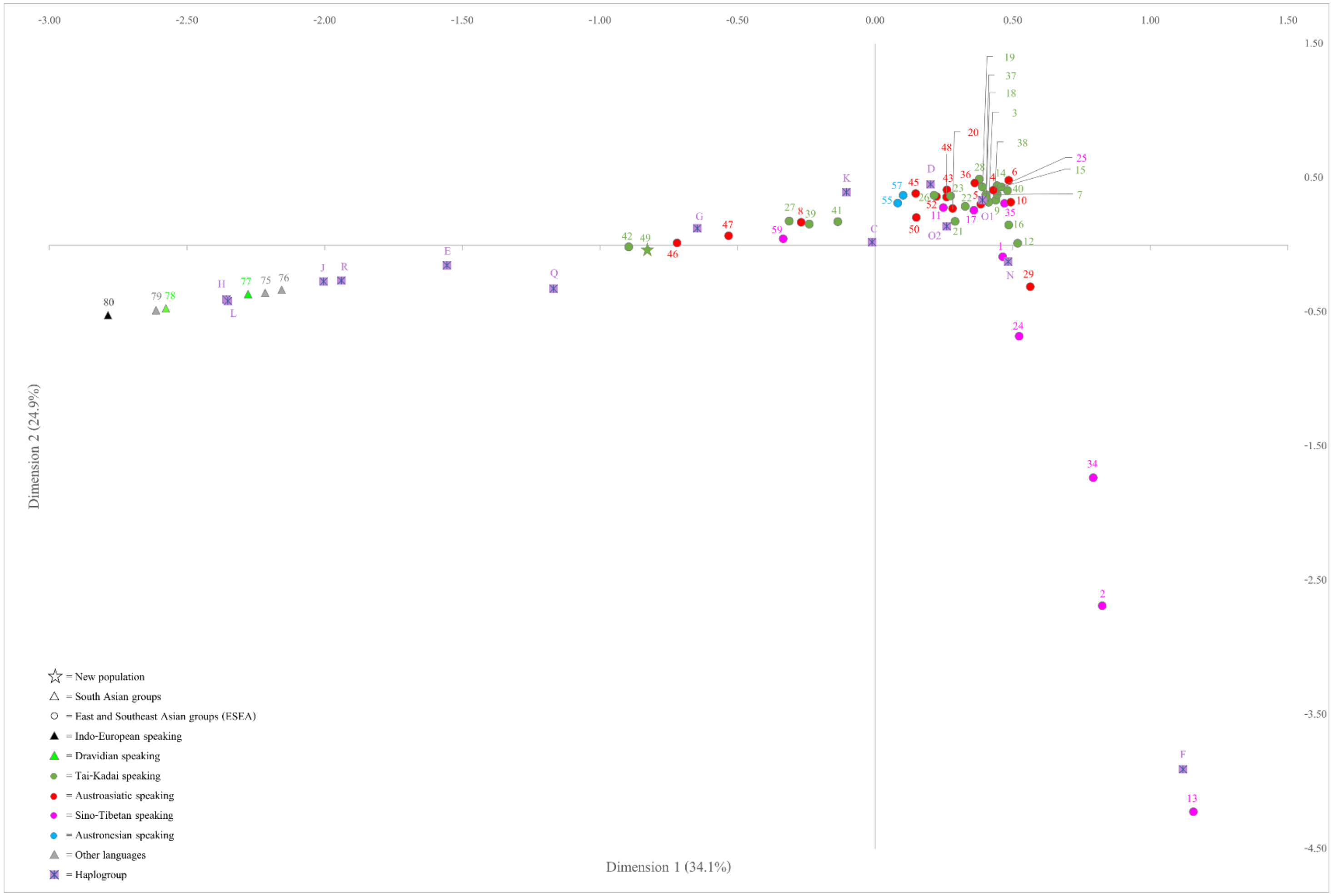

1 = Lisu_T
2 = Lahu_T
3 = Khonmueang
4 = Blang
5 = Palaung
6 = Khmu
7 = Lue
8 = Mon_N
9 = Khuen
10 = Lawa
11 = Karen
12 = Tay
13 = Phula
14 = Nung
15 = Lachi
16 = Thai
17 = Southern Han
18 = Dai
19 = BlackTai
20 = Kihn
21 = Phuan
22 = Shan
23 = LaoIsan
24 = Hani
25 = Sila
26 = Kaluang
27 = CentralThai_E
28 = Nyaw
29 = Sao
34 = Lahu_V

34 = Lahu_V
35 = Lolo
36 = HtinPray
37 = Laotian
38 = Phutai
39 = CentralThai_C
40 = Gelao
41 = CentralThai_W
42 = CentralThai_N
43 = Khmer_T7
45 = Bru
46 = Mon_C
47 = Mon_W
48 = Mon_NE
49 = SouthernThai_TK
50 = Nyahkur
52 = Suay
55 = Jarai
57 = Rhade
59 = Burmese
75 = SouthAsian1
76 = SouthAsian2
77 = Punjabi
78 = IndianTalugu
79 = SriLankan
80 = Bengali
